# Modeling of Experimental Data Supports HIV Reactivation from Latency after Treatment Interruption on Average Once Every 5–8 Days

**DOI:** 10.1371/journal.ppat.1005740

**Published:** 2016-08-25

**Authors:** Mykola Pinkevych, Stephen J. Kent, Martin Tolstrup, Sharon R. Lewin, David A. Cooper, Ole S. Søgaard, Thomas A. Rasmussen, Anthony D. Kelleher, Deborah Cromer, Miles P. Davenport

**Affiliations:** 1 Kirby Institute for Infection and Immunity, UNSW Australia, Sydney, Australia; 2 Department of Microbiology and Immunology, The University of Melbourne, Melbourne, Australia; 3 The Peter Doherty Institute for Infection and Immunity, The University of Melbourne and Royal Melbourne Hospital, Melbourne, Australia; 4 Department of Infectious Diseases, Alfred Hospital and Monash University, Melbourne, Australia; 5 Department of Infectious Diseases, Aarhus University Hospital, Aarhus, Denmark; University of North Carolina at Chapel Hill, UNITED STATES

Hill et al. provide a critique[1] of our recent paper, in which we developed two novel methods for estimating the rate of reactivation from latency directly from existing clinical data [2]. Our goal was to use minimal assumptions and be guided by the data. We found that across four cohorts, the best-fit frequency of HIV reactivation from latency was once every 5–8 days. Hill et al.’s modeling demonstrates an alternative approach, in which they start with a fixed rate of reactivation (four events per day, derived from modeling of time to drug resistance under therapy [3,4]) and then adjust the model and other parameters to fit their fixed reactivation rate. Importantly, amongst the various fitting described by Hill et al., they never actually fit the reactivation rate, instead always fixing it at their preferred value and adjusting the model and fitting other parameters around it. Hill et al. state that the data can be fit with “modest variation” of the reactivation rate. However, the parameters used require that ≈8% of patients reactivate less than once every 6 days (the mean rate we estimated in [2]) and, similarly, ≈8% reactivate more than 100 times per day (see S1 Methods). Below, we show that once the reactivation rate is fitted to all datasets, the results strongly support that the median frequency of HIV reactivation from latency is once every 5–8 days.

The first criticism by Hill et al. is that we did not incorporate multiple reactivation events or a distribution of reactivation rates into our model. They use simulation to incorporate such a distribution because of “lack [of] an analytical expression for the model” of multiple reactivation events. In response, we now derive an analytical approximation incorporating multiple reactivation events and apply it to all four available datasets (see S1 Methods). In each case, we fitted reactivation rate and initial viral load (*V*_*0*_), plus the distribution in reactivation rate. Because viral growth is a measureable parameter, we fixed this in the model, firstly using Hill et al.’s chosen viral growth rate (0.4/day) and then using a more realistic value obtained directly from the data (0.8/day) (see S1 Methods). Fig 1 shows fitting to all four datasets of (i) our original two-parameter model (panels A–D), (ii) the model with multiple reactivation events using Hill et al.’s preferred low growth rate (E–H), and (iii) a model with a more realistic growth rate (I–L). We note that only in 1 out of 12 fits (cohort 3 with a low growth rate, panel G, Fig 2) did we obtain a reactivation rate more frequent than once every 2 days. In all cases with realistic growth rates, the estimated rate of HIV reactivation from latency was very similar to our original model (once every 5–8 days) even once a distribution was included (Fig 1I–1L; Fig 2). Thus, the results presented by Hill et al. are highly dependent on the choice of a low viral growth rate and require an extremely wide distribution in reactivation rates.

**Fig 1:**
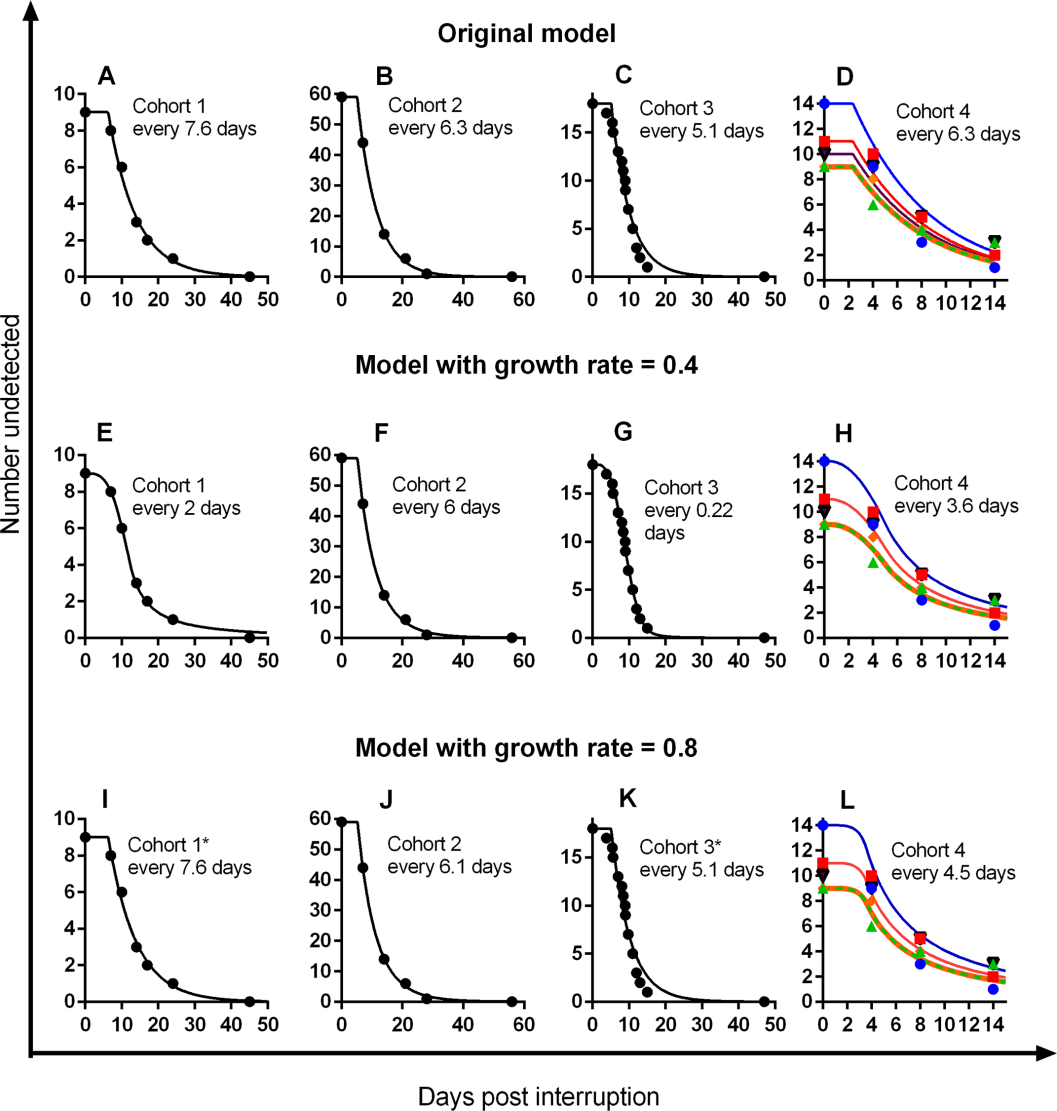
Estimating HIV reactivation rate using different models. The figure compares the fitting of the best-fit reactivation rate to each of the four cohorts using the original simple two-parameter model (panels A–D) as well as the more complex model proposed by Hill et al. that incorporates multiple reactivation events and a distribution in reactivation rates. The latter is fitted using a washout time of 0 days and either Hill et al.’s proposed slow viral growth rate (*g* = 0.4/day) (panels E–H) or a more realistic growth rate estimated from cohort 1 (*g* = 0.8/day) (panels I–L). In each case, the initial viral load (*V*_*0*_) and the mean and standard deviation of the reactivation rate is fitted. The average time between reactivation events is indicated for each cohort. Only for cohort 3, with a low growth rate (panel g), is estimated reactivation rate more frequent than once every 2 days. *Note: although a distribution in reactivation rates was fitted, the best fit model had a standard deviation of zero.

**Fig 2:**
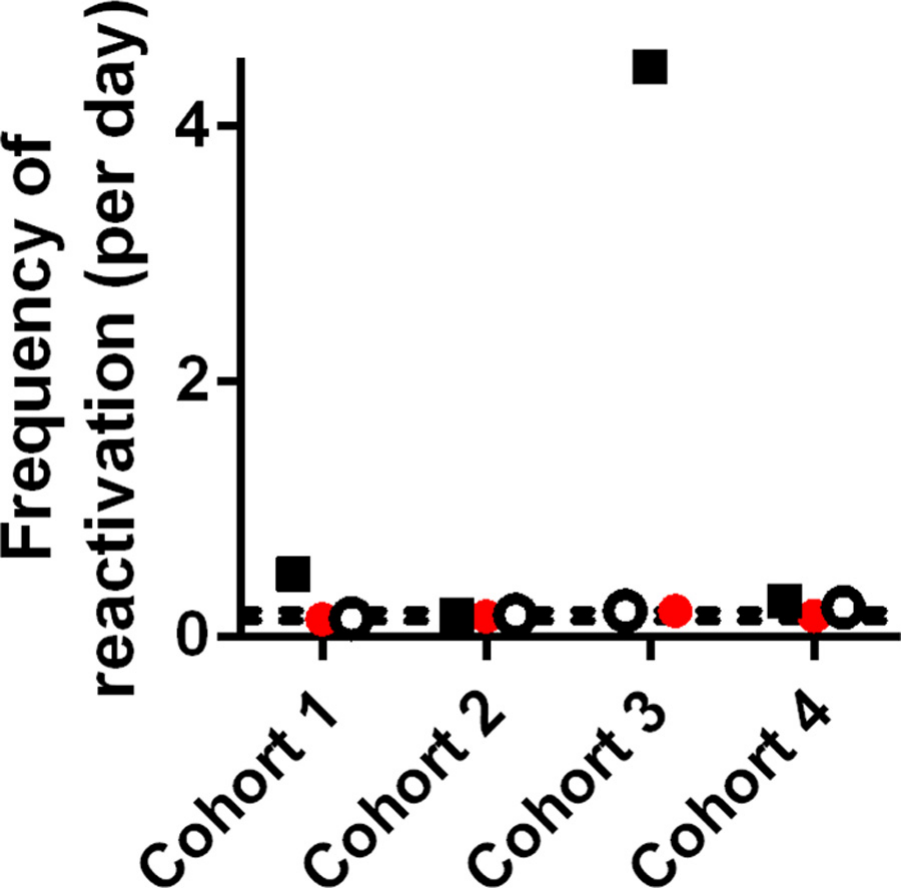
Frequency of HIV reactivation from latency estimated using different models: The mean frequency of reactivation estimated using the original model (red circles, corresponding to panels A–D of Fig 1), Hill’s model with slow growth rate (black squares, panels E–H of Fig 1), or Hill’s model with growth rate estimated from cohort 1 (open black circles, I–L of Fig 1) is shown. The frequency of reactivation estimated in Pinkevych et al. [2] is shown as dashed lines.

Hill et al. raise additional arguments about distributions in viral growth rate. In our previous publication, we found no significant correlation between growth rate and time-to-detection. We agree with Hill et al. that there is always a risk of a type II error in such analyses. However, the simulation performed by Hill et al. significantly misrepresents the probability of this error. Stating they observed no significant correlation (*p* > 0.05) in 50% of simulations grossly overstates the probability of observing the correlation seen in the data. The correlation between growth rate and time-to-detection in cohort 3 was in fact positive (*r* = 0.029, *p* = 0.9) despite an expected negative correlation. Thus, it is more appropriate to ask either, “In what proportion of simulations did we see an *r* value of 0.029 or greater?” or, “In what proportion of simulations was the *p* value greater than 0.9?” Using the parameters suggested in Hill et al.’s Fig 1D [1], we find that less than 2% of simulations were as poorly correlated as the observed data. Thus, we maintain our view that although growth rate must affect time-to-detection, based on the data this contribution is likely small.

Hill et al.’s primary argument is that there is strong prior evidence of high reactivation rates, and they cite four papers in support (references 1, 3–5 in their comment). None of the papers they cite in support of their reactivation rate of four-per-day actually analyses data on “reactivation from latency after treatment interruption” (the title of our earlier publication [2]). Two of these papers [3,5] base their estimates on time-to-drug-resistance under therapy, using a wide variety of assumptions on HIV replication under therapy, mutation rates, and drug selection. The third paper [6] measures T cell activation, not latent cell reactivation, so it does not provide an estimate. The fourth estimate comes from Hill et al.’s own previous work[4] but is derived directly from the estimate of reference [3] (see S1 Methods). Thus, the evidence for a reactivation rate of 4 per day hinges upon reference [3]’s use of a 13-parameter model of time to drug resistance, in which many of the input parameters are described as “roughly based on the literature”[3]. Hill et al. also argue that reactivation must be high because of a lack of correlation between reservoir size and rebound time in the literature. This appears to ignore recent publications showing such a correlation [7–9] as well as the apparent wide variation between different measures of reservoir size from peripheral blood [10].

Hill et al. have gone to some lengths to try to show that their previous estimate of HIV reactivation of 4 times a day could be made compatible with the data from the cohorts presented in Pinkevych et al. However, the failure to actually fit the key parameter significantly undermines these arguments. Once rigorous quantitative fitting is applied, the parameters suggested by Hill et al. do not provide the best fit to the data. Indeed, the additional analyses undertaken herein provide strong support for our previous estimate that HIV reactivates from latency approximately once every 5–8 days.

The derivation of the analytical approximation and a detailed explanation of the modeling are provided in S1 Methods because of word limits on this reply.

## Supporting Information

S1 Methods Supplementary methods including additional figures and derivation of the analytical expression. (DOCX)

S1 Data Raw data on time-to-detection across the four cohorts. (PDF)

## References

[1] Hill AL, Rosenbloom DIS, Siliciano JD, Siliciano RF. Insufficient evidence for rare activation of latent HIV in the absence of reservoir-reducing interventions. PLoS Pathog. 2016 10.1371/journal.ppat.1005679 PMC4999146 27560936

[2] Pinkevych M, Cromer D, Tolstrup M, Grimm AJ, Cooper DA, Lewin SR, et al HIV Reactivation from Latency after Treatment Interruption Occurs on Average Every 5–8 Days—Implications for HIV Remission. PLoS Pathog. 2015;11(7):e1005000 10.1371/journal.ppat.1005000 26133551 PMC4489624

[3] Pennings PS. Standing genetic variation and the evolution of drug resistance in HIV. PLoS Comput Biol. 2012;8(6):e1002527 10.1371/journal.pcbi.1002527 22685388 PMC3369920

[4] Hill AL, Rosenbloom DIS, Fu F, Nowak MA, Siliciano RF. Predicting the outcomes of treatment to eradicate the latent reservoir for HIV-1. Proceedings of the National Academy of Sciences. 2014;111(43):13475–80. 10.1073/pnas.1406663111 PMC4169952 25097264

[5] Rosenbloom DIS, Hill AL, Rabi SA, Siliciano RF, Nowak MA. Antiretroviral dynamics determines HIV evolution and predicts therapy outcome. Nat Med. 2012;18(9):1378–85. 22941277 10.1038/nm.2892 PMC3490032

[6] Ribeiro RM, Mohri H, Ho DD, Perelson AS. In vivo dynamics of T cell activation, proliferation, and death in HIV-1 infection: why are CD4+ but not CD8+ T cells depleted? Proc Natl Acad Sci USA. 2002;99(24):15572–7. 12434018 10.1073/pnas.242358099 PMC137758

[7] Li JZ, Etemad B, Ahmed H, Aga E, Bosch RJ, Mellors JW, et al The size of the expressed HIV reservoir predicts timing of viral rebound after treatment interruption. AIDS. 2016;30(3):343–53. 10.1097/QAD.0000000000000953 26588174 PMC4840470

[8] Assoumou L, Weiss L, Piketty C, Burgard M, Melard A, Girard P-M, et al A low HIV-DNA level in PBMCs at antiretroviral treatment interruption predicts a higher probability of maintaining viral control. AIDS. 2015;29(15):2003–7. 10.1097/QAD.0000000000000734 26355572

[9] Williams JP, Hurst J, Stöhr W, Robinson N, Brown H, Fisher M, et al HIV-1 DNA predicts disease progression and post-treatment virological control. Elife (Cambridge). 2014;3:e03821. 10.7554/eLife.03821 PMC4199415 25217531

[10] Eriksson S, Graf EH, Dahl V, Strain MC, Yukl SA, Lysenko ES, et al Comparative analysis of measures of viral reservoirs in HIV-1 eradication studies. PLoS Pathog. 2013;9(2):e1003174 10.1371/journal.ppat.1003174 23459007 PMC3573107

