# Supplementary material for: Modeling of Experimental Data Supports HIV Reactivation from Latency after Treatment Interruption on Average Once Every 5–8 Days
Source: PLoS Pathog. 2016 Aug 25;12(8):e1005740. doi: 10.1371/journal.ppat.1005740 (PMC4999223; doi:10.1371/journal.ppat.1005740)
Supplement: S1 Methods — (DOCX) [file ppat.1005740.s001.docx]

**Supplementary Methods to:**

***Modeling of experimental data supports HIV reactivation from latency after treatment interruption on average once every 5-8 days.***

**Mykola Pinkevych, Stephen J Kent**, **Martin Tolstrup, Sharon R Lewin, David A. Cooper, Ole S Søgaard, Thomas A. Rasmussen, Anthony D. Kelleher, Deborah Cromer and Miles P. Davenport**

**Modeling HIV reactivation from latency using time-to-detection of virus:**

*Modeling multiple reactivation events:*

*(with a single average reactivation rate)*

Hill *et al* point out that our previous model^1^ did not take into account the possibility that, at high reactivation rates, multiple reactivation founders could reactivate close to each other and thus the total viral load will grow faster than expected (shortening the time-to-detection)( Figure S1a-c). Hill *et al* state that due to the “*lack of an analytical expression for the model when multiple reactivation cells contribute to rebound*” they based their arguments upon simulation.

To resolve these issues, we have derived an approximation for the effect of multiple reactivating strains, as below.

We assume that reactivations occur in average every 1/*k* days, and the initial viral load of each reactivation is *V*_0_. Thus the viral load at time *t* after the first reactivation, that happened at time *t*_0_, can be expressed by formula:

$V(t)=V_{0}e^{g(t-t_{0})}+V_{0}e^{g(t-t_{0}-1/k)}+V_{0}e^{g(t-t_{0}-2/k)}+...+V_{0}e^{g(t-t_{0}-(n-1)/k)}, n=\left\lfloor(t- t_{0})k\right\rfloor+1$ . (1)

Applying the formula for the sum of terms for the geometric series , we can reduce the function (1) to form (2)

$V(t)=V_{0}e^{g(t-t_{0})}\frac{1-e^{-g/k(\left\lfloor(t-t_{0})k\right\rfloor+1)}}{1-e^{-g/k}}$ , (2)

where $\left\lfloor x\right\rfloor$ is the largest integer not greater then *x.*

The function in equation (2) is what we might see in one individual, where an integer number of reactivation events have occurred. However, across a population, the probability of a given number of reactivations is relevant, therefore we removed the discontinuities in (2) by substituting $\left\lfloor(t-t_{0})k\right\rfloor$ with $(t-t_{0})k$. After this substitution formula (2) can be reduced as it shown below


. (3)

Now we can find the delay between the first reactivation and detection of virus, putting *V*(*t*)=*T*, the detection threshold.

 . (4)

Thus, the time to detection curve in a homogeneous group will have form:

$\mathrm{P}(t)=P_{0}(1-\mathrm{F}_{\exp}(k,t-\Theta(k,g)),$ (5)

where *g* is the growth rate, *V*_0_  is the initial concentration of virus, P(*t*) is the number of people with undetectable virus at time *t*, *P*_0_ is the initial number of people, F_exp_(*k*, *t*) is cumulative density function of exponential distribution with the frequency *k*. These models were fitted in Wolfram Mathematica 9.1 (Wolfram Research inc., Champaign, Illinois, USA) using the function *NonlinearModelFit* and in GraphPad Prism (GraphPad Software, Inc. CA, USA). We used both an F-test and a corrected AIC to compare the models.

In our published model (Figure S2a and reference ^1^), we considered two parameters – the delay until the first viral detection *t_0_* (combining the drug washout time and the time for the virus to grow to the detection threshold), and the subsequent average frequency of viral reactivation, *k*.

*Comparing our original model with one incorporating multiple reactivation events.*

The model incorporating multiple reactivation events (equation 5) requires consideration of additional parameters, including washout time, initial viral load, frequency of reactivation, and growth rate of virus (+/- distribution in this reactivation rate). Consistent with the approach adopted by Hill *et al*, we fix the washout time (0 days) and viral growth rate (0.8 per day), and fit initial viral level, *V_0_* and the reactivation rate, *k.* (we later add a distribution in reactivation rates)

When we fit equation 5 to the data (ie: accounting for multiple reactivation events, but without a distribution in reactivation events) and compared it to our original fitting, this did not significantly change our estimates of reactivation rate (Figure S2).

Upon reflection it is clear why consideration of multiple reactivation events affects the analysis of average reactivation rates very little; When multiple reactivation events are considered, it can cause total viral load to reach detection earlier than expected, because multiple ‘bursts’ of virus ‘add up’ (Figure S1). For example, Hill et al suggest that at very high reactivation rates the time-to-rebound will be determined by *T*_wash_ + ln(*V*_R_/*kV*_0_)/*g*. Multiple reactivation events are thus most important when there is both a high reactivation rate and a low growth rate. When modeling an *average* reactivation rate, the impact of multiple reactivation events is also unimportant; since the ‘real’ initial viral load (*V_0_*) is unknown, the contribution of multiple founders arriving together and effectively elevating *V_0_* can always be compensated for by a higher fitted estimate of *V_0_*. However, when modeling a distribution of reactivation rates, then the effect of multiple reactivation rates becomes important, because it elevates the effective *V_0_* in those with the highest reactivation rates, causing them to reach the threshold for detection of virus earlier.

*Modeling a distribution in reactivation rates.*

Hill et al propose that a distribution in reactivation rates is important to consider. Thus, we adapted our approximation to take into account a distribution in reactivation rates;

$\mathrm{P}(t)=\frac{P_{0}(1-\int_{k_{\min}}^{k_{\max}} \mathrm{F}_{\exp}(k,t-\Theta(k,g))\mathrm{f}_{\text{lognorm}}(\mu,\sigma,k) dk)}{(\mathrm{F}_{\text{lognorm}}(\mu,\sigma,k_{\max})-\mathrm{F}_{\text{lognorm}}(\mu,\sigma,k_{\min}))}$ (6)

where f_lognorm_ (*μ,σ*,*k*) is the probability density function of the lognormal distribution with parameters *μ* and *σ* , *k*_min_ , *k*_max_ are the limits of integration.

*Repeating the analysis of Hill et al, but fitting reactivation rate.*

Hill et al simulate reactivation, and test different values for initial viral load and standard deviation of reactivation rate. However, they do not fit reactivation rate, and instead always keep it fixed at four reactivations per day.

We repeated the analysis proposed by Hill et al, using our analytical expression (equation 6). We first performed this using Hill et al’s preferred growth rate (0.4 per day), and fitting *V_0_*, and the mean and distribution of the reactivation rate (ie: parameters *μ* and *σ*, with median = *e^μ^* )^[[1]](#footnote-1)^. As can be seen from figure 1g of main text, the estimated median reactivation rate for cohort 3 is very similar to the rate they estimate (≈4 per day). However, for the other three cohorts the reactivation rate is much lower – 0.17- 0.5 per day (ie: one reactivation event every 2-6 days).

Hill et al chose to force the model to have a median reactivation rate of 4 events per day, and then adjust other parameters to fit this mean. This requires very wide distributions in reactivation rate. For example, Figure S3 shows the assumed distribution in reactivation rates for individuals in cohorts 1 and 4 (from figure S1c of their comment). It is noteworthy that this fitting requires that ≈8% of patients have a reactivation rate less than once every six days (the mean we estimated in our previous study). Moreover, ≈8% of patients have a reactivation rate >100 per day (which is hard to reconcile with a *V_0_* of 0.7 RNA copies/ml). It is clear that if the reactivation rate is fitted rather than fixed, and the model applied to all four datasets, the results do not appear to support Hill et al’s argument for a reactivation rate of four per day, even using their preferred growth rate.

*Best estimate of viral growth rate:*

Hill *et al* assume a low viral growth rate (0.4 per day), based on studies where sampling was performed weekly, or including all viral loads during the interruption^2-4^ (although viral growth slows near the peak, so including late viral loads inevitably leads to low estimates of growth^5,6^). However, it is clear from cohort 1 that when viral load is measured regularly (every 3-4 days), early viral growth rates are significantly higher, at ≈0.8 per day (see Figure S4). Studies in primary HIV infection have also shown that frequent early sampling provides better estimates of viral growth rate (which in primary infection is around 1.1 per day) ^5^, because viral growth slows over time as it becomes higher. Since the distribution in time-to-detection is influenced by viral growth rate when virus is at very low levels (ie: below the threshold of detection), we feel the growth rate from the earliest and most frequently sampled data is most representative. However, for comparison with Hill et al’s analysis we show results for both growth rates (ie: 0.4 per day, and 0.8 per day, see figure 1 e-l in main text).

*Modeling reactivation with a growth rate of 0.8 per day*

We repeated the analysis of all cohorts using the same fitting (equation 6) and what we believe is a much more realistic early growth rate for the virus (0.8 per day). In this case, the estimated reactivation rates ranged from once every 5-8 days, consistent with those reported in our original study. Even cohort 3, which showed a high reactivation rate when slow growth was used (figure 1g of main text), showed a reactivation rate of once every 5.1 days when a more realistic growth rate was used.

The results presented by Hill at al thus seem entirely dependent on the assumption of a low viral growth rate.

*Should models include a distribution in reactivation rates?*

Hill et al argue that including more parameters – particularly a distribution in reactivation rates – is required for the model fit. Our initial model fitted the data with only two parameters – a minimum delay until first detection, and a constant (mean) frequency of HIV reactivation from latency after this. Hill *et al* claim we ignored the possibility of variation in the frequency of reactivation amongst patients. However, in our paper we explicitly stated; “*it seems likely that the frequency of reactivation we estimate for the cohorts represents an average frequency in the cohort, and there will be a distribution amongst individuals*” (see paragraph 2 of discussion in ^1^). In addition, we analyzed the data on sequential interruptions of patients in cohort 4 and explicitly showed that there was a distribution in reactivation rates amongst patients (also in discussion of ^1^). That is, we did not ignore the possibility of a distribution in reactivation rates – we showed it exists.

Although we agree that there is likely a distribution in reactivation rates in the population, the question for modeling is whether including this distribution actually improves the model fit. Hill et al state that ‘only two model parameters are identifiable’ and ‘the choice of which were fixed and which were fit was arbitrary’. That is, they acknowledge that they are over-fitting the data, but feel that including parameters based upon belief is justified. We used an F-test to compare the fits of models with and without the distribution in reactivation rates (Figure S5), and found that inclusion of the distribution of reactivation rates does not improve the fit. Therefore we prefer to estimate the rates based on a mean reactivation, even though we previously showed there was a distribution, because we cannot accurately estimate the distribution from the data.

**Weak prior evidence for high reactivation rates.**

Using their models with ≥4 parameters, Hill *et al* observe that many different parameter combinations can be used to fit the data. Somewhat surprisingly, instead of concluding that the rate of HIV reactivation is not able to be estimated using their approach, Hill *et al* argue for a high frequency of HIV reactivation from latency (4-5 reactivations per day). Hill *et al* cite both Pennings^7^ and Hill *et al* ^8^ as sources of the estimated rate of 4 – 5 reactivation events per day. As we observe in the main comment, Pennings based her calculation on a 13-parameter model fit to a single outcome being the time to evolution of drug resistance. Some of these many input parameters included assumptions about mutation, selection etc, and were stated as ‘roughly based on literature’ ^7^. Thus, this analysis was not directed at estimating the frequency of reactivation following treatment interruption, and did not analyse data relevant to this question.

Hill *et al* ^8^ both misinterpret Pennings’ rate, and also use the estimate from Pennings as a key means of parameterizing their own model (See section 2.4 of the supporting information of reference ^8^ and analysis below). Close inspection of Hill *et al*’s paper^8^ shows that their estimate of 4 reactivation events per day depends directly on a parameter *N_LAT_* (number of cells reactivating per generation) from Pennings. The rate of reactivation from latency (*k*) calculated by Hill *et al* can be shown to be the convergent solution of the following iterative equation

$k_{n+1}=\frac{AR_{0}(1-e^{-\frac{kn}{A}(R_{0}+\frac{A}{N_{LAT}}-1)})}{R_{0}+\frac{A}{N_{LAT}}-1}$ (7)

where *A* and *R0* are as described in Hill *et al* ^8^ (*A* = 57cells/day and *R0* = 1.4) and *NLAT* is taken directly from Pennings^7^. Thus, it is clear that *k* depends directly on *A, R0* and *NLAT* only. Figure S6 below highlights the dependence and shows how the Hill estimate of reactivation rate would vary as the parameter *N_LAT_* from Pennings is changed.

In addition, Hill *et al* misquote Pennings’ estimate as five reactivations per day, rather than the reported five reactivations per generation (and 200 generations per year, equating to 2.7 reactivations per day^7^). Using the correct parameter from Pennings (and the dependence of the Hill estimate on the Pennings estimate) would have resulted in an estimated reactivation rate of 1.9/day.

**Ratios of reactivation founder virus:**

Hill et al raise a third argument, based on frequencies of reactivation founder virus (figure 2 of their comment). They argue that the distribution in reactivation founder clone sizes within an individual can be explained by distributions in growth rates of the different clones with the individual. They suggest that this would require a standard deviation in growth rate of 0.19 per day. This is very similar to the distribution in growth rate between individuals assumed in figure 1b of their comment (0.2 per day). However, although the similarity in these distributions may seem superficially reassuring, there is an underlying problem with the distribution in growth rate *between individuals* and the distribution in viral growth rate *within individuals*. That is, at high reactivation frequency (as proposed by Hill et al), the growth rate observed in an individual will converge on the growth rate of the fastest growing clone. So, across individuals, we observe the fastest growing clone only. It is difficult to envisage a distribution of growth rates within individuals on the scale proposed that can also produce the distribution proposed between individuals.

**Future work:**

Our analysis of the data is our best attempt to fit rates from the available data. We hope others will take up the challenge of refining the models and applying them to other datasets. In particular, an exact model of the impact of multiple reactivation events to replace our approximation would be helpful (even though at the estimated reactivation rates we find, this will have minimal impact on the overall effect). We provide the raw data used in our modeling in the accompanying supplementary data file.





**Figure S1:** **Schematic of the impact of multiple reactivation events on time-to-detection of virus at high, medium, and low reactivation rates.**





**Figure S2: Comparing models with and without multiple reactivation events.**

In order to test whether including the effects of multiple reactivation events affects the estimated frequency of reactivation of HIV after treatment interruption, we compare model fits to four patient cohorts. The top panel (a-d) shows fitting of a model of a delay plus frequency of reactivation (2 parameters, as used in ^1^). The best-fit average frequency of reactivation is shown. The bottom panel (e-h) shows the best-fit to a model incorporating multiple reactivation events, with a mean reactivation rate across the cohort (ie: it did not include a distribution in reactivation rates). The model fitted both initial viral load (*V_0_*) and reactivation rate. The best-fit frequencies of reactivation are shown.


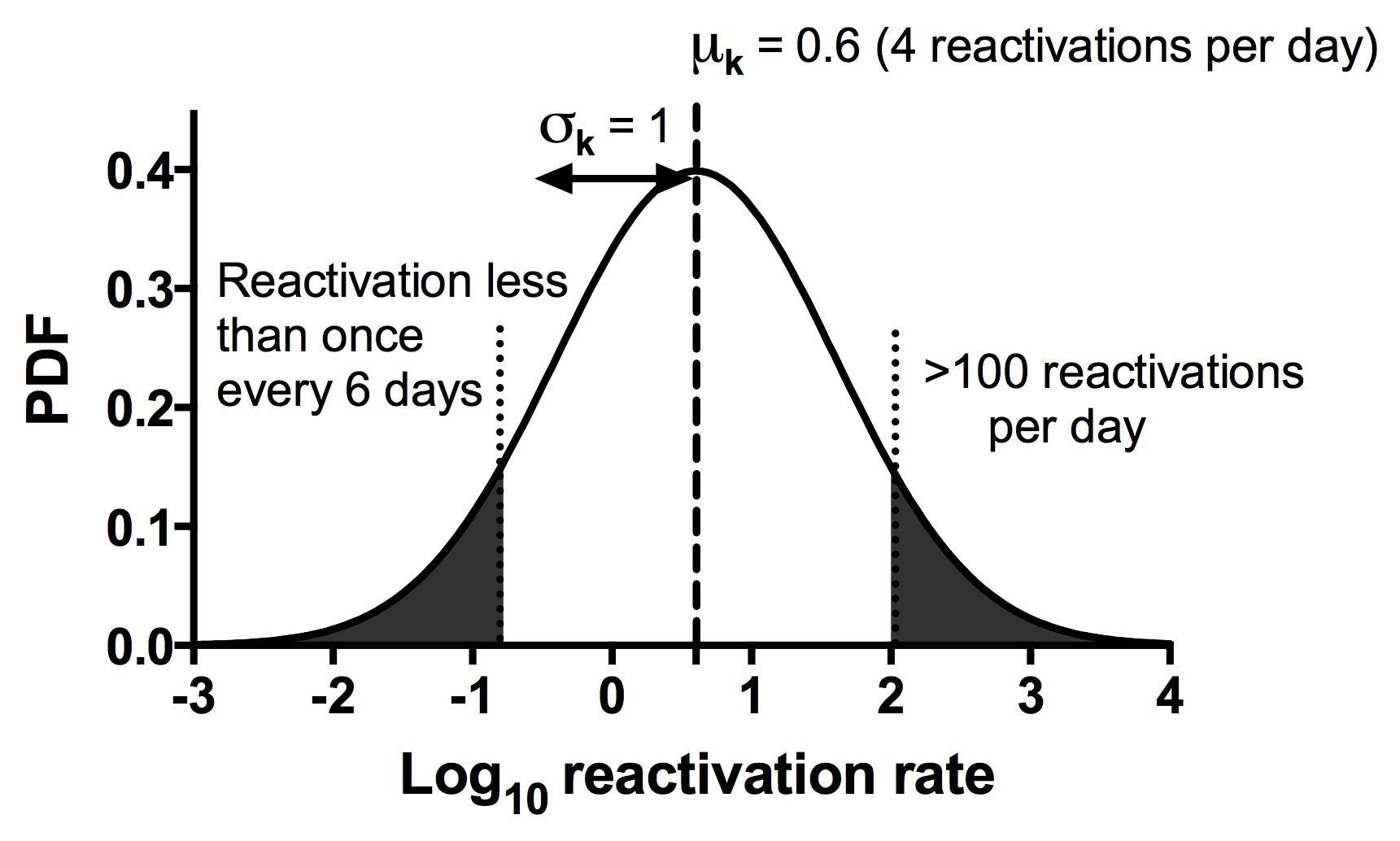


**Figure S3: Assumed distribution of reactivation events.**

The distribution in the frequency of reactivation events across the population simulated by Hill *et al* for cohorts 1 and 4. As described, the distribution is log_10_-normal with a mean of 0.6 (=4 reactivation events per day), and a standard deviation of 1 log_10_.





**Figure S4: HIV viral growth rates from cohort 1 of reference ^1^.** (A) Growth rates were estimated from serial viral loads of individual patients (data shown in symbols, best-fit slopes for individuals as thin colored lines). The mean growth rate for the cohort of 0.8 per day is shown as a thick solid red line. A growth rate of 0.4 per day is shown as a red dashed line for comparison. (B) The distribution in growth rates for the cohort, with median indicated. A growth rate of 0.4 per day is indicated as a dashed line..





**Figure S5: A distribution in reactivation rates does not provide a better fit to the data.**

We compared two models of HIV reactivation either considering only a mean reactivation rate (panels a-d) or considering a log-normal distribution of reactivation rates as proposed by Hill *et al*. In each case we assumed no washout time, and a viral growth rate of 0.8 / day. We then fitted the initial viral load and mean (or mean and distribution) of reactivation rates. P-values comparing the simple (top) and more complex (bottom) models are shown (using F-test, as these are nested models). In all cases, the more complex model does not provide a significantly better fit than the simple model. Moreover, in most cases the estimated distributions of reactivation rate were narrow.

*For cohort 1 and 3, the fit with σ = 0 provided the best fit.


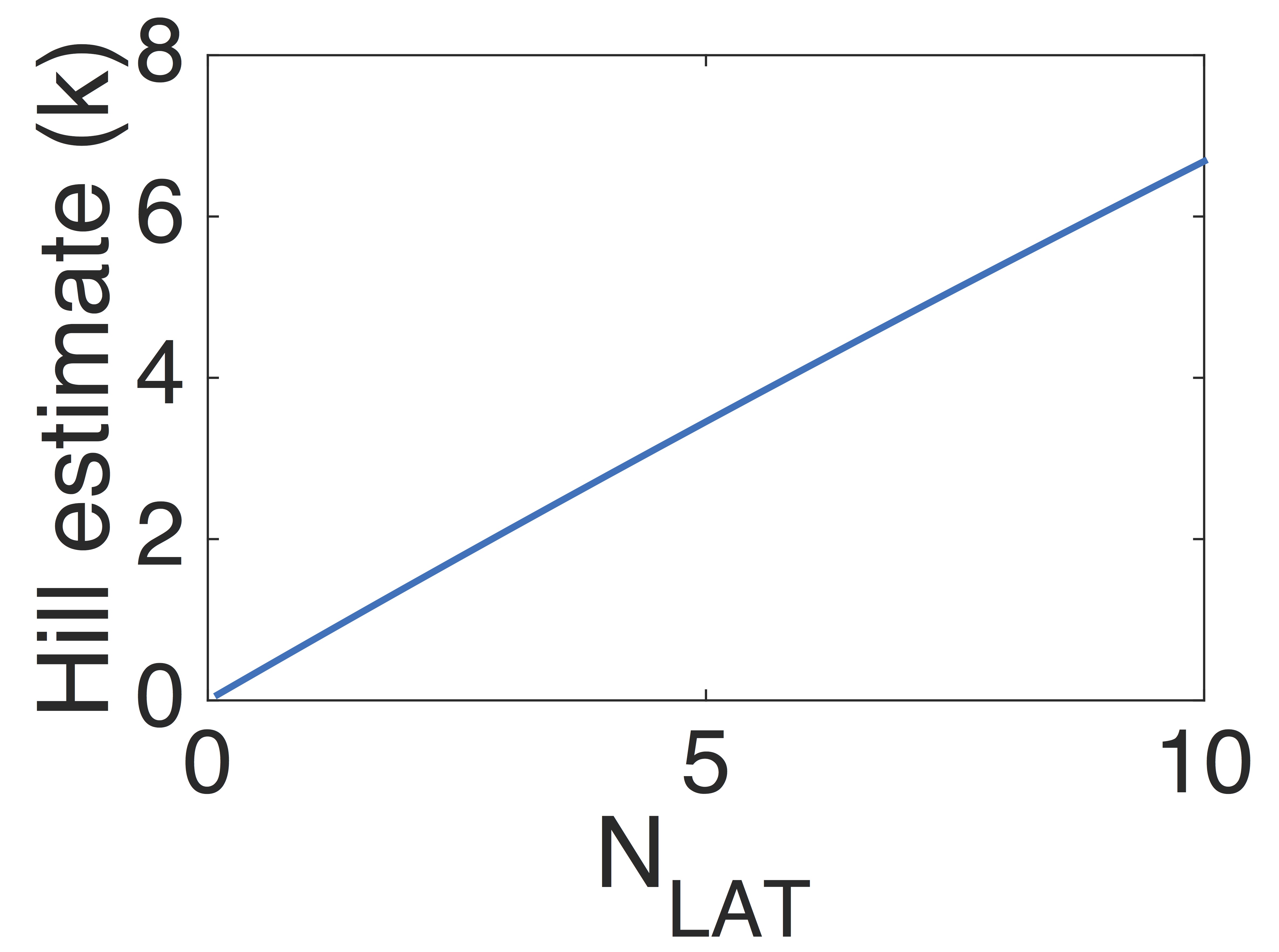


Figure S6: Dependence of reactivation rate parameter (*k*) from Hill *et al* (reference^8^) on *N_LAT_* parameter from Pennings et. al. (reference ^7^). Note that this uses the same (incorrect) assumption as Hill et al used that *N_LAT_* is cells per day, and not cells per generation ^8^.

**References:**

1 Pinkevych, M. *et al.* in *PLoS Pathog* Vol. 11 e1005000 (2015).

2 Ruiz, L. *et al.* in *AIDS* Vol. 14 397-403 (2000).

3 Ruiz, L. *et al.* in *AIDS* Vol. 15 F19-27 (2001).

4 Luo, R., Piovoso, M. J., Martinez-Picado, J. & Zurakowski, R. in *PLoS ONE* Vol. 7 e40198 (2012).

5 Ribeiro, R. M. *et al.* in *J Virol* Vol. 84 6096-6102 (2010).

6 Cromer, D., Tey, S. K., Khanna, R. & Davenport, M. P. in *J Virol* Vol. 87 3376-3381 (2013).

7 Pennings, P. S. in *PLoS Comput Biol* Vol. 8 e1002527 (2012).

8 Hill, A. L., Rosenbloom, D. I. S., Fu, F., Nowak, M. A. & Siliciano, R. F. in *Proceedings of the National Academy of Sciences* (2014).

1. Note that we express lognormal parameters here in base *e*, not base 10 as reported by Hill et al. [↑](#footnote-ref-1)
