# Supplementary material for: Modeling of Experimental Data Supports HIV Reactivation from Latency after Treatment Interruption on Average Once Every 5–8 Days
Source: PLoS Pathog. 2016 Aug 25;12(8):e1005740. doi: 10.1371/journal.ppat.1005740 (PMC4999223; doi:10.1371/journal.ppat.1005740)
Supplement: S1 Data — (PDF) [file ppat.1005740.s002.pdf]

## Data on time to detection across cohorts.

**Cohort 1**

| | | | | | | | | | |
|---|---|---|---|---|---|---|---|---|---|
| **Patient ID** | 1 | 2 | 3 | 4 | 5 | 6 | 7 | 8 | 9 |
| **Days to detection** | 10 | 14 | 45 | 14 | 24 | 7 | 17 | 10 | 14 |

**Cohort 2**

| | | | | | | | | | | | | | | | | | | |
|---|---|---|---|---|---|---|---|---|---|---|---|---|---|---|---|---|---|---|
| **Patient ID** | 1 | 2 | 3 | 4 | 5 | 6 | 7 | 8 | 9 | 10 | 11 | 12 | 13 | 14 | 15 | 16 | 17 | 18 |
| **Days to detection** | 21 | 21 | 7 | 7 | 14 | 14 | 14 | 14 | 7 | 28 | 7 | 7 | 14 | 7 | 14 | 14 | 14 | 7 |
| **Patient ID** | 19 | 20 | 21 | 22 | 23 | 24 | 25 | 26 | 27 | 28 | 29 | 30 | 31 | 32 | 33 | 34 | 35 | 36 |
| **Days to detection** | 21 | 7 | 14 | 21 | 14 | 7 | 7 | 14 | 14 | 7 | 21 | 7 | 14 | 14 | 14 | 14 | 14 | 7 |
| **Patient ID** | 37 | 38 | 39 | 40 | 41 | 42 | 43 | 44 | 45 | 46 | 47 | 48 | 49 | 50 | 51 | 52 | 53 | 54 |
| **Days to detection** | 21 | 7 | 14 | 21 | 14 | 7 | 14 | 14 | 14 | 28 | 28 | 14 | 14 | 14 | 14 | 28 | 14 | 14 |
| **Patient ID** | 55 | 56 | 57 | 58 | 59 | | | | | | | | | | | | | |
| **Days to detection** | 14 | 21 | 56 | 14 | 28 | | | | | | | | | | | | | |

**Cohort 3**

| | | | | | | | | | | | | | | | | | | |
|---|---|---|---|---|---|---|---|---|---|---|---|---|---|---|---|---|---|---|
| **Patient ID** | 1 | 2 | 3 | 4 | 5 | 6 | 7 | 8 | 9 | 10 | 11 | 12 | 13 | 14 | 15 | 16 | 17 | 18 |
| **Days to detection** | 5.4 | 3.8 | 8.9 | 11 | 47 | 8.4 | 9 | 9.8 | 5.6 | 12 | 9.8 | 11 | 8 | 15 | 13 | 12 | 7 | 7 |

**Cohort 4**

| | | | | | | | | | | | | | | |
|---|---|---|---|---|---|---|---|---|---|---|---|---|---|---|
| **Patient ID** | 102 | 104 | 105 | 109 | 111 | 112 | 116 | 118 | 120 | 121 | 125 | 127 | 128 | 130 |
| ***Interruption 1*** | | | | | | | | | | | | | | |
| **Days to event** | 4 | 8 | 8 | 8 | 8 | 4 | 4 | 4 | 14 | 8 | 4 | 14 | 8 | 14 |
| **Event** | positive | positive | positive | positive | positive | positive | positive | positive | positive | positive | positive | positive | positive | censored |
| ***Interruption 2*** | | | | | | | | | | | | | | |
| **Days to event** | 8 | | 14 | 4 | 8 | 14 | 8 | 14 | 14 | 4 | | 8 | 8 | 14 |
| **Event** | positive | | censored | positive | positive | positive | positive | positive | positive | censored | | positive | positive | censored |
| ***Interruption 3*** | | | | | | | | | | | | | | |
| **Days to event** | 8 | | 14 | 4 | 4 | 4 | | | 14 | 4 | 14 | 4 | 8 | 14 |
| **Event** | positive | | censored | positive | positive | censored | | | censored | censored | positive | positive | positive | censored |
| ***Interruption 4*** | | | | | | | | | | | | | | |
| **Days to event** | 8 | | 14 | | 8 | 8 | 8 | | 14 | 8 | 14 | | 4 | 14 |
| **Event** | positive | | censored | | positive | positive | positive | | positive | censored | positive | | positive | censored |
| ***Interruption 5*** | | | | | | | | | | | | | | |
| **Days to event** | 8 | | 14 | 8 | 14 | 8 | 4 | 14 | 14 | 8 | 4 | | | 14 |
| **Event** | positive | | censored | positive | positive | positive | positive | positive | censored | positive | censored | | | censored |
